# Does Tumor Size Improve the Accuracy of Prognostic Predictions in Node-Negative Gastric Cancer (pT1-4aN0M0 Stage)?

**DOI:** 10.1371/journal.pone.0101061

**Published:** 2014-07-08

**Authors:** Mu Xu, Chang-Ming Huang, Chao-Hui Zheng, Ping Li, Jian-Wei Xie, Jia-Bin Wang, Jian-Xian Lin, Jun Lu

**Affiliations:** Department of Gastric Surgery, Fujian Medical University Union Hospital, Fuzhou, Fujian Province, China; University Hospital Heidelberg, Germany

## Abstract

**Background:**

The prognostic importance of tumor size in gastric cancer is unclear. This study investigated whether the inclusion of tumor size could improve prognostic accuracy in node-negative gastric cancer.

**Methods:**

Clinical and pathological data of 492 patients with node-negative gastric cancer who underwent radical surgery in our department from January 1995 to December 2008 were analyzed. The prognostic accuracy of T stage was compared with that of T stage plus tumor size. The ability of tumor size to improve the 95% confidence interval (CI) of postoperative 5-year survival rate in gastric cancer patients was assessed. Different T stages plus tumor size were further analyzed to assess improvements in prognosis.

**Results:**

Mean tumor size was 3.79±1.98 cm with a normal distribution. Multivariate analysis showed that tumor size and T stage were independent prognostic factors. Postoperative 5-year survival rate tended to decrease as tumor size increased in 1 cm increments. The addition of tumor size to T stage improved accuracy in predicting 5-year survival by 4.2% (P<0.05), as well as improving the 95% CI of postoperative 5-year survival rate by 3.2–5.1%. The addition of tumor size improved the predictive accuracy of postoperative 5-year survival rate by 3.9% (95% CI 70.4%–91.1%, P = 0.033) in patients with stage T3N0M0 tumors and by 6.5% (95% CI 68.7%–88.4%, P = 0.014) in patients with stage T4aN0M0 tumors.

**Conclusions:**

Tumor size is an independent prognostic factor for survival in patients with node-negative gastric cancer, as well as improving prognostic accuracy in stage T3/4aN0M0 tumors.

## Introduction

Gastric cancer is a common gastrointestinal malignancy in China and the second most common cause of cancer-related deaths worldwide [1], [2]. Lymph node metastasis remains one of the most important predictors of survival following curative resection in gastric cancer [3]–[5]. Although overall survival is better in patients with node-negative than node-positive gastric cancer, a significant number of the former still develop recurrence [6], [7]. Identifying the prognostic factors associated with improved outcomes in patients with node-negative gastric cancer is therefore important. Although depth of tumor invasion [7]–[10] and lymphovascular invasion [7], [8] have been shown to be prognostic in these patients, the prognostic significance of tumor size is still uncertain. Tumor size can be measured easily without special tools, and in some cancers, such as breast and lung cancer, tumor size is included in the tumor-node-metastasis (TNM) staging system [11]. To assess the prognosis impact of tumor size on patient survival, we retrospectively analyzed outcomes in 492 patients with node-negative gastric cancer.

## Patients and Methods

This study involved a prospectively collected database of patients who underwent radical gastrectomy for gastric cancer at the Department of Gastric Surgery, Affiliated Union Hospital of Fujian Medical University, Fuzhou, China, from January 1995 to December 2008. A total of 1586 consecutive and nonselected gastric cancer patients underwent lymphadenectomy, with more than 15 lymph nodes examined in each patient. After excluding 1094 node-positive patients, we analyzed the remaining 492 patients with node-negative gastric cancer. Their clinical and histopathologic data was collected and recorded using a specifically designed data collection form. Lymph nodes were meticulously dissected from the en bloc specimens, and the classification of the dissected lymph nodes was determined by specialized surgeons who reviewed the excised specimens after surgery based on the Japanese Classification of Gastric Carcinoma (JCGC) [12]. Based on the 7th Edition of UICC TNM system [13], T categories were defined as: T1 (tumor invades mucosa), T2 (tumor invades muscularis propria), T3 (tumor invades subserosa), and T4a (tumor penetrates serosa without invasion of adjacent structure).

Patients were included if they underwent curative (R0) resection, defined as no macroscopically or microscopically residual tumor, with no less than D2 lymph node dissection, and pathologic examination of resected specimens. In addition, none of these patients had received neoadjuvant chemotherapy, and all had complete medical records. Patients with gastric stump cancer, infiltration of surrounding organs (T4b) or distant metastases (hepatic, lung, peritoneal dissemination, or extraregional lymph nodes such as the retropancreatic, mesenteric, and para-aortic lymph nodes) were excluded.

### Measurement of tumor diameter

**Figure 1:**
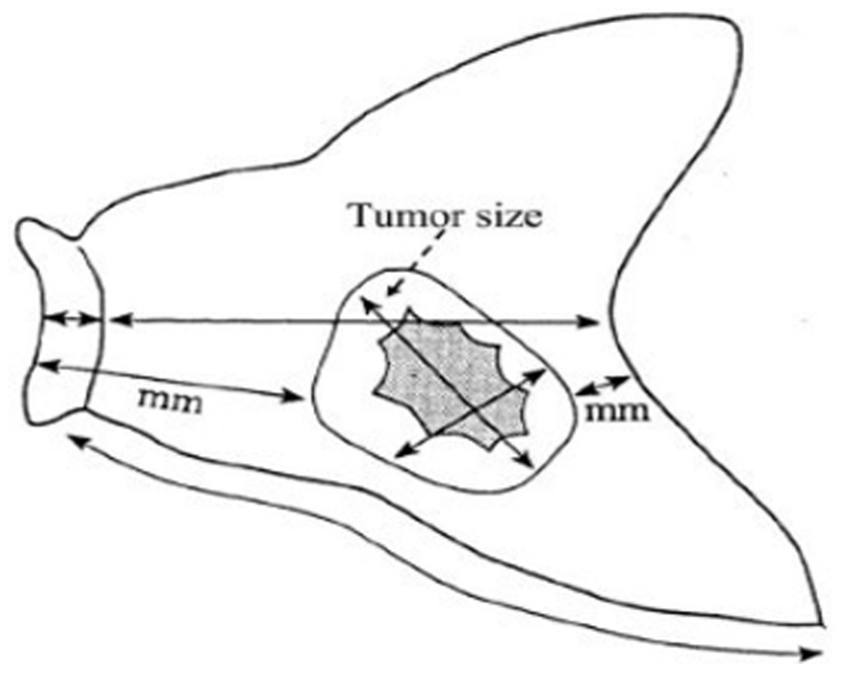
The resected stomach (mucosal side).

Tumor size was measured according to the JCGC [12]. Briefly, the resected stomach was opened along the greater curvature so the whole mucosa could be observed. If the tumor was located on the greater curvature, the stomach was opened in some other way, along the lesser curvature. The opened stomach was placed on a flat board with the mucosal side up and examined macroscopically. The lengths of the greater and lesser curvature, as well as the attached portion of the esophagus and/or the duodenum and the size and thickness of the tumor, were recorded (Fig. 1). The longer tumor size was used in the current study [12]. The distance between the tumor border and both the proximal and distal cut ends were also recorded. When tumor margin was unclear, the resected stomach was fixed by formalin for 1 hour. Afterwards, the tumor margin was determined by the pathologists with gross observation of the microscopic examination [12], [14].

### Ethics Statement

Ethics committee of Fujian union hospital approved this retrospective study. Written consent was given by the patients for their information to be stored in the hospital database and used for research.

### Follow-up

Routine follow-up consisted of physical examination, laboratory tests (including measurements of CEA, CA19-9 and CA125 concentrations), chest radiography, abdominopelvic ultrasonography and computed tomography (CT). Patients were followed-up every 3 months during the first year and every 6 or 12 months thereafter, for a total of 5 years. Endoscopy was performed every 6 or 12 months. All surviving patients were followed for more than five years. Overall survival (OS) was calculated from the date of diagnosis to last contact, date of death, or date when the survival information was collected.

### Statistical analysis

All statistical analyses were performed with the Statistical Package for Social Science (SPSS) version 18.0 for Windows. The χ^2^ test was used to evaluate the difference in proportions, and Student's t-tests were used to evaluate continuous variables. Multivariate analysis was performed using the Cox proportional hazards model in order to further evaluate all of the significant prognostic factors which were found in the univariate analysis. Survival analysis was performed using the Kaplan-Meier method and curves were compared with the log-rank test. Finally, T stages were complemented with tumor size in multivariate Cox regression models addressing cancer-specific survival. Predictive accuracy estimates were compared between models according to whether they included tumor size. The confidence interval (CI) method was used to compare the difference in means between predictive accuracy estimates for models that either included or did not include tumor size. In other words, T stages were analysized alone to assess the accuracy in predicting 5-year overall survival rate by Cox regression models. Then, tumor size was added in the model together with T stages to estimate whether the predictive accuracy was improved so as to evaluate the impact of tumor size on the predictive accuracy in node-negative gastric cancer. The difference between two analyses was the improvement in prediction. The difference was also analyzed according to different T stages. Each model was subjected to bootstrap resampling for internal validation and to reduce overfit bias. All p values were two-sided, with p values<0.05 considered statistically significant.

## Results

### Distribution of tumor size

**Figure 2:**
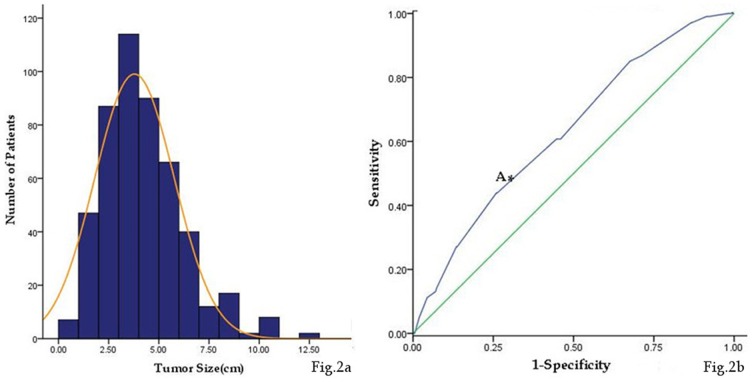
Distribution and the cutoff value of tumor size. (a) Histogram of the number of patients with regard to tumor size. (b) Receiver- operating characteristic curve (A: Shows sensitivity and specificity for a tumor size cutoff value of 4.75 cm were 53.9% and 73.2%, respectively).

Tumor size was normally distributed (P = 0.611) with a mean of 3.79±1.98 cm (Fig. 2a). Receiver operating characteristic (ROC) analysis indicated that a cutoff value of 4.75 cm yielded a sensitivity of 53.9% and a specificity of 73.2% in predicting survival after gastric surgery (AUC = 0.730, 95% CI 0.573–0.689, P = 0.000) (Fig. 2b).

### Results of postoperative follow-up

**Figure 3:**
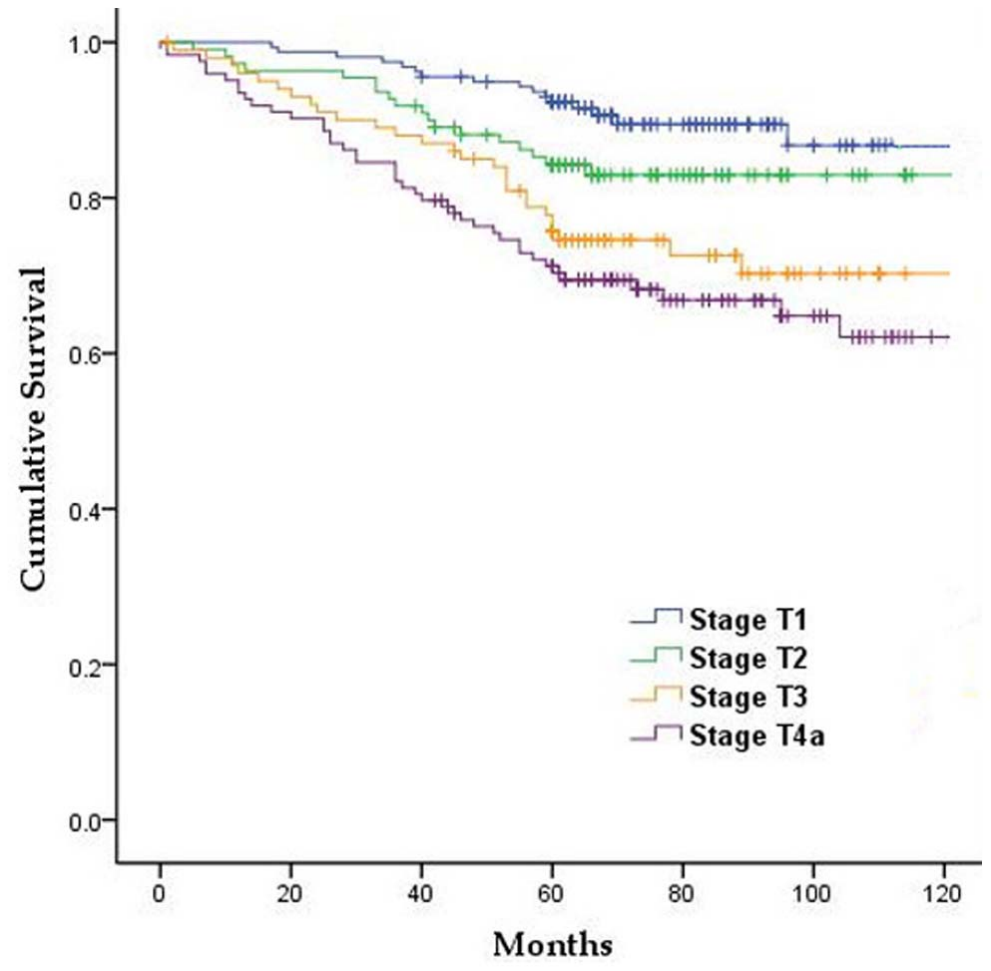
Survival curves of patients based on T stage.

Of the 492 patients, 448 (91.1%) were followed-up. The 5-year OS rate of all patients was 81.9%. The 5-year survival rates of patients with stage pT1, pT2, pT3 and pT4a tumors were 92.3%, 84.2%, 75.7%, and 71.2%, respectively, with these differences being statistically significant (P<0.05, Fig. 3).

### Univariate and multivariate survival analysis for all patients

Univariate analysis showed that tumor size (P = 0.000), depth of invasion (P = 0.000), and Borrmann type (P = 0.026) were significantly associated with 5-year OS rate. In contrast, gender (P = 0.758), age (P = 0.257), distribution of tumor location (P = 0.100), histological type (P = 0.908), and type of resection (P = 0.740) were not associated with survival (Table 1). Multivariate analysis using a Cox proportional hazards model showed that tumor size (P = 0.022) and depth of tumor invasion (P = 0.006) were independent predictors of poor patient prognosis (Table 2).

**Table 1:** Univariate analysis of patients by Kaplan-Meier method.

Variables	Case(n)	5-year survival (%)	*χ* ^2^	P value
**Gender**			0.095	0.758
Male	381	81.4		
Female	111	83.5		
**Age(yr)**			1.284	0.257
<60	255	84.2		
≥60	237	79.4		
**Tumor size(cm)**			13.586	0.000
<4.75	345	85.6		
≥4.75	147	73.2		
**Depth of invasion**			23.14	0.000
T1	158	92.3		
T2	110	84.2		
T3	101	75.5		
T4a	123	71.2		
**Location**			6.245	0.100
Upper third	147	79.4		
Middle third	77	84.3		
Lower third	228	84.8		
Diffuse#	40	70.0		
**Borrmann type**			4.979	0.026
I/II	238	86.0		
III/IV	254	78.0		
**Histologic type** †			0.013	0.908
Differentiated	231	82.9		
Undifferentiated	261	81.0		
**Operation type** *			0.110	0.740
Total	231	81.7		
Subtotal	261	82.0		

# Diffuse, the location of tumor was more than two areas.

† Graded according to Japanese Classification of Gastric Carcinoma.

Differentiated, papillary, or well/moderately differentiated tubular adenocarcinoma;

Undifferentiated, poorly differentiated, or mucinous adenocarcinoma or signet-ring cell carcinoma.

*Total, total gastrectomy;

Subtotal, subtotal gastrectomy (including proximal subtotal gastrectomy and distal subtotal gastrectomy).

**Table 2:** Prognostic factors retained at multivariate analysis by Cox model.

Variables	B	SE	Wald	Df	Sig.	Exp(B)	95.0% CI*
**Tumor size(cm)**	0.072	0.055	1.698	1	0.022	1.074	1.065–1.397
**Depth of invasion**	0.294	0.106	7.667	1	0.006	1.342	1.090–1.653
**Borrmann type**	0.186	0.208	0.0798	1	0.372	1.205	0.801–1.812

*95%CI, 95% Confidence interval.

### Correlation between 5-year OS rate and tumor size according to 1 cm intervals

**Figure 4:**
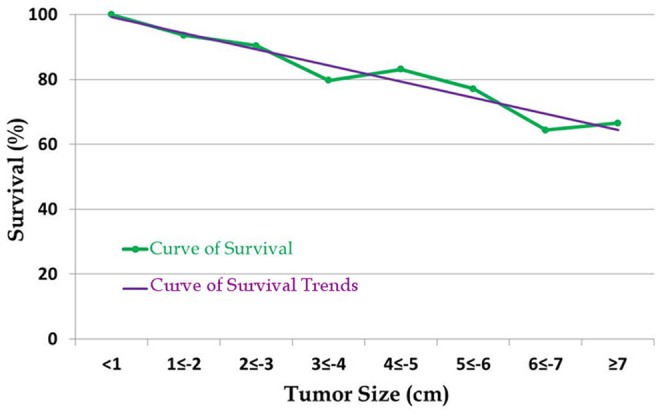
Distribution of survival by 1 cm tumor size intervals.

Patients were divided into eight groups according to 1 cm tumor size intervals. We found that the 5-year OS rate tended to decrease as tumor size increased, with 5-year OS rates in these eight groups of 100%, 93.6%, 90.4%, 79.7%, 83.1%, 77.1%, 64.4%, and 66.6% respectively (Fig. 4).

### Impact of T stage on the accuracy of prognosis with and without tumor size

Cox regression analysis showed that the accuracy of T stage alone in predicting 5-year OS rate was 72.2% (95% CI 66.3%–78.1%), whereas the addition of tumor size to T stage increased the accuracy to 76.4%(95% CI 70.4%–82.4%), with a 4.2% (95% CI 3.2%–5.1%, P<0.05) increase in accuracy (Table 3).

**Table 3:** Univariate and multivariate Cox regression models predicting depth of invasion, tumor size and 5-year survival in patients with gastric cancer.

Factor	5-year survival		
	Univariate		Multivariate(T+S)†
	HR*	Predictive accuracy	HR
	P value		P value
	(95% CI#)		(95% CI)
**T stage(T)**	-	0.722	-
T2 vs. T1	0.305		0.396
	0.000		0.006
	(1.177–3.525)		(1.205–1.763)
T3 vs. T1	0.473		0.549
	0.007		0.044
	(2.274–4.815)		(1.307–2.983)
T4a vs. T1	0.779		0.841
**s**	0.010		0.037
	(1.482–4.261)		(1.514–3.376)
**Tumor size(S)**	1.188	0.710	1.082
	0.000		0.049
	(1.097–1.287)		(1.972–3.204)
**Predictive accuracy of the model**	-	-	0.764(+0.042)

† T, T Stage; S, Tumor Size.

*HR, Hazard Ratio.

# 95%CI, 95% Confidence interval.

### Accuracy of prediction of combinations of T stage and tumor size

Further analysis of combinations of T stage and tumor size showed that the addition of tumor size significantly improved the accuracy of prediction in patients with stage T3/4aN0M0 gastric cancer (Table 4).

**Table 4:** Effects of T stage and tumor size on the accuracy of prognostic predictions in patients with gastric cancer.

T stage	Improvement in Prediction (%)	95%CI*	P value
**T1**	1.2	48.2%–82.1%	0.124
**T2**	1.9	52.1%–86.5%	0.079
**T3**	3.9	70.4%–91.1%	0.033
**T4a**	6.5	68.7%–88.4%	0.014

*95%CI, 95% Confidence interval.

## Discussion

The key to improving the prognosis of patients with gastric cancer is to individualize treatment to maximize effectiveness in different patients. Identifying prognostic factors is helpful in finding patients at high risk of recurrence. Lymph node metastasis is one of the most important prognostic factors in gastric cancer after curative (R0) resection [3]–[5], with overall survival being significantly longer in node-negative than node-positive patients. However, some patients with node-negative gastric cancer experience recurrence and metastasis [6], which may result in fatal outcomes. The recurrence rate in patients with node-negative early gastric cancer was found to range from 1.7–3.4% [15]–[18]. Kooby et al. [7] showed it was important to confirm the prognostic factors which influenced the node-negative gastric cancer. Some of them with high risk factors such as micro-metastasis might result in recurrence. Therefore, it's of great significance to identify prognostic factors associated with poor survival in patients with lymph node-negative gastric cancer.

Depth of wall invasion [7]–[10] and lymphovascular invasion [7], [8] are among the most important indicators associated with survival in patients with node-negative gastric cancer. Moreover, lymphovascular invasion has been associated with increased recurrence rate, resulting in poorer prognosis [19]. In some cancers, such as breast and lung cancer, tumor size is a significant prognostic factor and is included in the tumor-node-metastasis (TNM) staging system [11]. Tumor size can be easily and objectively measured without the need for specific tools. However, the prognostic significance of tumor size in gastric cancer remains unclear. One study found that tumor size was a predictor of survival on univariate, but not multivariate, analysis [20], whereas other studies have reported that tumor size is an independent prognostic factor. For example, a study in which tumor size was divided into four subgroups, <2 cm, <3 cm, <5 cm and ≥5 cm, found that tumor size was independently predictive of survival [21]. Maximum tumor diameter >8 cm was associated with significantly poorer OS than maximum diameter <8 cm [22]. Errors may occur, however, when classifying tumor size without considering the effects of invasion depth and lymph node metastasis on tumor size [23]. It is difficult to identify the most important prognostic factors because many variables are interrelated. The impact of tumor size on prognosis can be precisely evaluated only when depth of invasion and lymph node metastasis are specified. Thus, to eliminate the effects of lymph node metastasis, we analyzed the effects of tumor size on prognosis in patients with node-negative gastric cancer. In agreement with previous findings [24], we found that tumor size was independently prognostic of patient survival, as was the depth of wall invasion. As tumor size increased, the 5-year OS rate tended to descend. Larger tumor size was associated with greater invasion of tissue surrounding the stomach. Moreover, infiltration of the serosa increased tumor contact with the peritoneal cavity and the likelihood of free cancer cells in the peritoneal cavity. We also assessed whether the addition of tumor size could improve prognosis in patients assorted by T stage using multivariate Cox regression models. We found that inclusion of tumor size increased prediction accuracy by 4.2%. On the other hand, it also proved that tumor size influenced the prognosis of node-negative gastric cancer and improved the accuracy of prognostic prediction.

We also performed a subgroup analysis based on the depth of wall invasion to eliminate the effect of T stage on tumor size. We found that the accuracy of T stage in predicting 5-year OS was significantly improved when tumor size was included in patients with stage T3/4aN0M0 gastric cancer. Shallow wall invasion, especially in patients with early gastric cancer located in the mucosa or submucosa, was less likely to be associated with distant metastasis, regardless of tumor size, thus limiting tumor spread in such patients. As a result, curative resection of these tumors would also completely resect any micrometastases, reducing the postoperative recurrence rate and improving patient prognosis. Tumor size therefore had less impact on prognosis in patients with stage T1/2N0M0 tumors. Tumors that penetrated over the submucosa, however, would be more likely to be in contract with the lymph vessels, since most of the latter are located in this layer. Interactions between tumors and lymphatic tissue would likely increase with increasing tumor size. Therefore, as tumor size increased, so would the probability of micro-metastases migrating from the tumor through the lymph vessels [7]–[8], increasing the postoperative recurrence rate increase and resulting in poorer prognosis. Moreover, tumors which penetrated over the submucosa would be more likely to invade the blood vessels, enhancing the likelihood of metastatic disease. So did the increase of neural invasion in these tumors. Cancer cells may breach the perineurium at sites of vascular ingrowth, offering another potential route of dissemination and leading to poorer prognosis [25]. For tumors that invaded the serosa, thus penetrating the gastric wall, tumor size was likely associated with a larger area of serosal invasion, increasing the likelihood of intraperitoneal dissemination and poorer prognosis. In addition, tumor stroma produces cytokines that modulate immune reactions, which are responsible for signal transduction and facilitate tumor invasiveness [26]–[28]. All of these factors increased the possibility of recurrence, leading to poorer prognosis. Therefore, tumor size had a stronger impact on prognosis in patients with stage T3/4aN0M0 than earlier stage gastric cancer.

In conclusion, we found that tumor size was a clinical predictor of survival in patients with node-negative gastric cancer. Tumor size may also be served as an assistant indicator and help predict prognosis in patients with stage T3/4aN0M0 gastric tumors.
